# Arterial stiffness, fatty liver and the presence of coronary artery calcium in a large population cohort

**DOI:** 10.1186/1475-2840-12-162

**Published:** 2013-11-05

**Authors:** Ki-Chul Sung, Young-Hyo Lim, Sungha Park, Seok-Min Kang, Jeong Bae Park, Byung-Jin Kim, Jin-Ho Shin

**Affiliations:** 1 Division of Cardiology, Department of Internal Medicine, Kangbuk Samsung Hospital, Sungkyunkwan University School of Medicine, #108, Pyung Dong, Jongro-Ku, Seoul 110-746, Republic of Korea; 2 Division of Cardiology, Department of Internal Medicine, College of Medicine, Hanyang University, Seoul, Republic of Korea; 3 Division of Cardiology, Severance Cardiovascular Hospital and Cardiovascular Research Institute, College of Medicine, Yonsei University, Seoul, Republic of Korea; 4 Division of Cardiology, Department of Internal Medicine, Cheil General Hospital, College of Medicine, Kwandong University, Seoul, Republic of Korea

**Keywords:** baPWV, Arterial stiffness, Coronary artery calcium (CAC) score, Atherosclerosis, Fatty liver

## Abstract

**Background:**

We tested whether fatty liver, brachial-ankle pulse wave velocity (baPWV) and conventional cardiovascular risk factors were associated with a coronary artery calcium (CAC) score > 0 (as a marker of the presence of early atherosclerosis) in a cohort of healthy Korean adults.

**Method:**

The study population consisted of individuals who underwent a comprehensive health examination in 2010 at Kangbuk Samsung Hospital, College of Medicine, Sungkyunkwan University in South Korea. The 6009 subjects of total 7371 participants who had an assigned CAC score following coronary computed tomography (CT) scanning and baPWV were analyzed.

**Results:**

Among the study subjects, 39.2% of the population had evidence of fatty liver by ultrasound and 4.6% of the population had evidence of CAC score > 0. Among individuals with a CAC score = 0, 38% of the individuals had fatty liver compared with 58% of the individuals with a CAC score > 0. The individuals with a CAC score > 0 also had higher blood pressure and had more metabolic abnormalities. The prevalence of CAC score > 0 was increased according to baPWV quartiles and was higher in the fatty liver group in comparison with those without fatty liver. The odds ratio for CAC score > 0, after adjusting for clinical risk factors, showed a significant elevation with increasing quartiles of baPWV and the presence of fatty liver.

**Conclusion:**

We showed that both fatty liver and baPWV are independently associated with the presence of CAC, a marker of preclinical atherosclerosis. These associations are independent of conventional risk factors and medical history.

## Introduction

Arterial stiffness has been shown to be an independent predictor of cardiovascular mortality and a marker of early detection of target organ damage by cardiovascular disease [1,2]. Pulse wave velocity (PWV) is a practical marker of arterial stiffness; brachial-ankle (ba) PWV has been widely used in clinical research in Korea and Japan [3-6]. baPWV is a relatively simple, noninvasive test that could be a useful adjunct to conventional coronary artery disease (CAD) risk factors in identifying patients and subgroups in the population at increased risk for cardiovascular events. Although arterial stiffness and CAD are closely related, the correlation between arterial stiffness and coronary artery calcium (CAC) score, an early marker of atherosclerosis, in the general population has not yet been studied [7-10].

Several prospective studies have reported an increased incidence of cardiovascular events in people with nonalcoholic fatty liver disease (NAFLD) [11-14]. However, it is still unclear whether NAFLD is simply a risk marker that is coexistant with other recognized cardiovascular disease (CVD) risk factors associated with insulin resistance or whether it is an independent cardiovascular risk factor on its own [15,16]. Fatty liver has been shown to be associated with CAC score [17]. CAC scoring with cardiac computed tomography (CT) is a sensitive method to demonstrate the presence of early atherosclerosis, and the use of CAC score may improve the prediction of cardiovascular (CV) risk in asymptomatic and high risk individuals [7,8,18-20].

Thus, analyzing the association between CAC score with NAFLD and arterial stiffness may provide insight into a better understanding of the relationship between NAFLD, arterial stiffness and atherosclerosis. Using the data from a retrospective cohort study with measurements of fatty liver, baPWV and CAC score together with conventional cardiovascular risk factors, we investigated the relationship between fatty liver, baPWV and a CAC score > 0. Thus, we tested whether fatty liver, baPWV and conventional risk factors of metabolic syndrome (MetS) (waist circumference, glucose, triglyceride, high density lipoprotein cholesterol [HDL-C], and blood pressure) were associated with a CAC score > 0 (as a marker of the presence of early atherosclerosis) in a population cohort in Korea.

## Methods

The study population consisted of individuals who underwent a comprehensive health examination in 2010 at Kangbuk Samsung Hospital, College of Medicine, Sungkyunkwan University in South Korea. The population included 7,371 participants who had an assigned CAC score following coronary CT scanning and PWV. The individuals were excluded from the current analyses if data were missing for the following variables: alcohol consumption (n = 38), smoking (n = 46), exercise (n = 37) and waist circumference (n = 7). Furthermore, to investigate healthy subjects without the influence of past medical history and medication, we excluded those who answered prior cerebrovascular accidents (n = 16), prior CAD (n = 62), prior diabetes (n = 254), prior hypertension (n = 835) and prior hepatitis or positive hepatitis B/C viral serologic marker (n = 269), leaving data of 6,009 participants for this analysis. The study was approved by the institutional review board at Kangbuk Samsung Hospital. Informed consent requirement was waived because personal identifying information was not accessed.

BMI was calculated as weight in kilograms divided by height in meters squared. Blood samples for laboratory examinations were collected after an overnight fast. Fasting plasma glucose, total cholesterol, triglyceride (TG) and HDL-C concentrations were measured using Bayer Reagent Packs on an automated chemistry analyzer (Advia 1650 Autoanalyzer; Bayer Diagnostics, Leverkusen, Germany). Low density lipoprotein cholesterol (LDL-C) concentration was calculated using direct measurement.

Questionnaires were used to ascertain information regarding alcohol consumption (glass/day), smoking (never, ex, current), and frequency of moderate activity per week. Moderate activity was defined as more than 30 minute of activity per day that induced slight breathlessness.

All computed tomography scans were obtained with a Lightspeed VCT XTe-64 slice MDCT scanner (GE Healthcare, Tokyo, Japan) with the same standard scanning protocol using 40*2.5-mm section collimation, 400 ms rotation time, 120 kV tube voltage, and 124 mAS (310 mA*0.4 second) tube current under ECG-gated dose modulation. Quantitative CAC scores were calculated according to the method described by Agatston et al. [14].

Bilateral brachial and ankle blood pressure, and arterial pulse waves were measured using an automatic wave analyzer VP-1000 (Omron Healthcare Co., Ltd, Kyoto, Japan). Individuals were examined in the supine position after at least 5 minute of bed rest. Bilateral brachial and posterior tibial artery pressure waveforms were recorded by an oscillometric method using the occlusion/sensing cuffs adapted to both arms and both ankles. baPWV was calculated for each arterial segment as the path length divided by the corresponding time interval. The coefficient of variation was 6.5% for baPWV.

Abdominal ultrasonography (Logic Q700 MR; GE, Milwaukee,WI, USA) using a 3.5 MHz probe was performed in all subjects by experienced clinical radiologists, and fatty liver was diagnosed, based on standard criteria, including hepatorenal echo contrast, liver brightness, and vascular blurring [21].

### Statistical analysis

Statistical analysis of the data was performed using SPSS version 15.0 (SPSS, Point Richmond, CA, USA). Continuous variables were expressed as mean ± SD for normally distributed variables or median (interquartile range) for other variables. baPWV was stratified into quartiles. Categorical variables were expressed as percentages and compared between groups using the χ2 test. Logistic regression was used to determine the odds ratio (OR) and 95% confidence intervals (CI) for the presence of a CAC score > 0 for the highest baPWV quartile compared with the lowest baPWV quartile as the reference. In these regression models, the following variables were entered to investigate the independence of the relationship between baPWV in the highest quartile and CAC scores: age, sex, smoking status (never/ ex or current), frequency of moderate activity per week (defined by ≥ 3 episodes of moderate activity of > 30 minute per day that induced slight breathlessness), LDL-C, TG, HDL-C, alcohol consumption (glass/day), waist circumference and fatty liver. We used logistic regression to determine the OR for the presence of a CAC score > 0 in individuals stratified by quartiles of baPWV and as a function of fatty liver.

## Results

**Figure 1:**
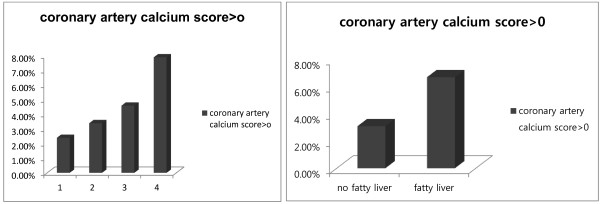
**The prevalence of CAC score > 0 2.4% in baPWV Quartile 1 (Q1), 3.4% in 2 (Q2), 4.6% in 3 (Q3), 7.9% in 4 (Q4) (*p *< 0.001) and 3.1% in no fatty liver group, 6.7% in fatty liver group.** (*p* < 0.001).

As shown in Table 1, all baseline characteristics except fasting blood glucose level were significantly different between individuals with a CAC score = 0 compared with those with a CAC score > 0. Among the study subjects, 39.5% of them had evidence of fatty liver by ultrasound and 4.5% had evidence of a CAC score > 0. Among individuals with a CAC score =0, 38.6% of the individuals had fatty liver compared with 59.0% in the individuals with a CAC score > 0. The individuals with a CAC score > 0 also had higher blood pressure and had more metabolic abnormalities including higher TG and LDL-C concentrations and lower HDL-C concentration. The prevalence of CAC score > 0 was increased according to baPWV quartiles and was higher in the fatty liver group compared with those with no fatty liver (Figure 1).

**Table 1:** Baseline characteristics according to CAC score = 0 and CAC score >0

	**CAC = 0**	**CAC > 0**	***p***
Subjects number	5736	273	
Age (years)	41.0 ± 6.2	47.2 ± 7.3	< 0.001
Sex, n (%)			< 0.001
Male	4626 (80.6%)	256 (93.8%)	
Female	1110 (19.4%)	17 (6.2%)	
Fasting glucose (mg/dl)	94.7 ± 11.0	95.0 ± 19.0	0.700
Triglyceride (mg/dl)	135.4 ± 92.6	151.6 ± 100.8	0.005
Median[interquartile]	113 [79,163]	130 [94,181]	
HDL- cholesterol (mg/dl)	52.9 ± 12.4	49.0 ± 11.0	< 0.001
LDL- cholesterol (mg/dl)	123.9 ± 31.9	137.8 ± 29.3	< 0.001
ALT (U/L)	27.3 ± 32.8	29.6 ± 19.5	< 0.001
AST (U/L)	24.0 ± 17.0	26.5 ± 16.3	< 0.001
BMI (kg/m^2^)	24.1 ± 3.0	24.8 ± 2.6	< 0.001
Waist circumference (cm)	83.9 ± 8.1	85.8 ± 6.8	< 0.001
Systolic BP (mmHg)	116.3 ± 12.0	119.1 ± 12.1	< 0.001
Diastolic BP (mmHg)	74.4 ± 8.6	76.6 ± 8.1	< 0.001
HR (beats/min)	64.5 ± 8.9	66.4 ± 9.0	< 0.001
Mean baPWV (cm/s)	1302.9 ± 146.3	1379.9 ± 174.8	< 0.001
Alcohol (glass/d)	1.6 ± 2.5	2.3 ± 3.3	< 0.001
Current smoker, n (%)	1254 (21.9%)	88 (32.2%)	<0.001
Physical activity (≥ 3/week), n (%)	1003 (17.5%)	62 (22.7%)	0.019
Fatty liver, n (%)	2215 (38.6%)	161 (59.0%)	<0.001

Data are mean±SD or median (interquartile range) unless otherwise noted.

*CAC* = coronary artery calcium; *HDL* = high-density lipoprotein; *LDL* = low-density lipoproteinl; *AST* = Aspartate Aminotransferase; *ALT* = Alanine Aminotransferase; *BMI* = body mass index; *BP* = blood pressure; *HR* = heart rate; *baPWV* = brachial ankle plse wave velocity.

Physical activity category defined by ≥ 3 episode of moderate activity of > 30 min/d that induced slight breathlessness.

In the multiple regression model adjusted for multiple cardiovascular risk factors, we investigated the OR for CAC score > 0 with increasing quartiles of baPWV controlled for the presence of fatty liver. Table 2 shows the OR for CAC score > 0, after adjustment for age, sex, glucose, TG, HDL-C, LDL-C, waist circumference, systolic blood pressure, alcohol consumption, smoking status, physical activity and fatty liver; the results showed a significantly increased in the highest quartile of baPWV.

**Table 2:** Associations between baPWV quartiles, fatty liver, cardio-metabolic risk factors, and CAC score > 0; derived from a multivariable logistic regression model containing all variables

	**Odds ratio (95% CI)**	***p***
Sex, female	0.309 (0.177-0.539)	< 0.001
Age, per year	1.140 (1.119-1.162)	< 0.001
Triglyceride, per mg/dl	0.999 (0.997-1.001)	0.367
HDL cholesterol, per mg/dl	0.979 (0.965-0.992)	0.002
LDL cholesterol, per mg/dl	1.009 (1.005-1.013)	< 0.001
Glucose, per mg/dl	0.985 (0.973-0.998)	0.021
Systolic BP, per mmHg	1.005 (0.992-1.017)	0.476
Alcohol, glass/d	1.035 (0.993-1.079)	0.104
Smoking (no versus ex or current)	0.958 (0.725-1.267)	0.765
Physical activity ≥ 3/week	1.315 (0.957-1.807)	0.091
Waist circumference per cm	0.983 (0.962-1.006)	0.145
Fatty liver	1.953 (1.450-2.632)	< 0.001
baPWV Quartile 1		
Quartile 2	1.014 (0.646-1.591)	0.952
Quartile 3	1.143 (0.736-1.774)	0.551
Quartile 4	1.627 (1.005-2.507)	0.027

*CAC score* = coronary artery calcium score; *HDL* = high-density lipoprotein; *LDL* = low-density lipoprotein; *BP* = blood pressure; *baPWV* = brachial ankle pulse wave velocity.

Next, we examined the prevalence (%) and adjusted OR of CAC score > 0 according to the presence of fatty liver, stratified by quartiles of baPWV (Table 3). In the lowest PWV quartile, there were 15 (1.4%) subjects with a CAC score > 0 were noted in 1084 subjects with no fatty liver compared to 20 (4.7%) of 422 subjects with a CAC score > 0 in the group with fatty liver (*p* < 0.001). In the group (of people in the baPWV 1^st^ quartile) with fatty liver compared with the group with no fatty liver, the adjusted OR (95% CI, *p* value) for CAC score > 0 was 1.86 (0.81 – 4.30, *p* = 0.145) (Model 4). However, in the 2^nd^ quartile there were 20 (2.2%) subjects with a CAC score > 0 in 921 subjects with no fatty liver, compared to 32 (5.4%) of 592 subjects with fatty liver (*p* < 0.001). The adjusted OR for CAC score > 0 was 2.80 (1.42 – 5.55, *p* = 0.003). In the highest baPWV quartile, even after adjusting for many kinds of variables (model 4) in healthy subjects, we identified a statistically significant increased OR, 1.90 (1.19 – 3.03, *p* = 0.007). Sensitivity analyses investigating the associations with higher levels of CAC score showed similar point estimates, but wider confidence intervals, as a consequence of there being only 147 people with CAC score > 20 and 40 people with CAC score > 100. After adjusting the same variables in Table 2, the OR for CAC score > 20 in the 2^nd^ quartile of baPWV is 1.43 (0.35 - 5.88, *p* = 0.35); in the 3^rd^ quartile, 1.55 (0.38 - 6.29, *p* = 0.54); in the 4^th^ quartile, 3.98 (1.08 - 14.6 *p* = 0.38); and the OR for fatty liver is 1.98 (0.94 - 4.16, *p* = 0.07). For CAC score > 100, the 2^nd^ quartile of baPWV is 0.91 (0.49 - 1.71, *p* = 0.77); in the 3^rd^ quartile, 0.99 (0.54 - 1.82, *p* = 0.90), in the 4^th^ quartile, 1.62 (0.90 - 2.91, *p* = 0.11); and the OR for fatty liver is 1.36 (0.92 - 2.01, *p* = 0.13). By adopting higher thresholds, there is decreased power to show the independent associations with baPWV or fatty liver with the CAC score, because there are fewer people with a CAC score > 20 or >100 compared to people with a CAC score > 0. However, it is important to note that (with wide 95% CIs) the B coefficients are very similar in the relationships between fatty liver or baPWV with the CAC score, regardless of whether the threshold is CAC > 0 or CAC score > 20. Thus, these data suggest that the strengths of the relationships between both fatty liver and baPWV with the CAC score are similar, regardless of the threshold of CAC score chosen for the analyses.

**Table 3:** Prevalence and odds ratio for the presence of coronary artery calcium according to baPWV quartile and fatty liver status

**baPWV quartile**	**Number (%)**		**Odds ratio (95% CI), *p***			
	**No fatty liver**	**Fatty liver**	**Model 1**	**Model 2**	**Model 3**	**Model 4**
Q1 < 1209 cm/s	15/1084 (1.4%)	20/422 (4.7%)	2.44 (1.21 - 4.90), 0.013	2.62 (1.26 - 5.44), 0.010	2.06 (0.92 - 4.62), 0.080	1.86 (0.81 - 4.30), 0.145
1209 cm/s ≤ Q2 < 1301 cm/s	20/921 (2.2%)	32/592 (5.4%)	2.15 (1.20 - 3.85), 0.010	2.25 (1.25 - 4.05), 0.007	2.26 (1.20 - 4.25), 0.012	2.80 (1.42 - 5.55), 0.003
1301 cm/s≤ Q3 <1394 cm/s	29/843 (3.4%)	39/651 (6.0%)	1.61 (0.97 - 2.68), 0.068	1.66 (0.99 - 2.78), 0.055	1.73 (1.00 - 3.02), 0.052	1.85 (1.03 - 3.34), 0.040
Q4 ≥ 1394 cm/s	48/785 (6.1%)	70/711 (9.8%)	2.04 (1.35 - 3.08), 0.001	2.04 (1.34 - 3.110), 0.001	1.74 (1.12 - 2.71), 0.015	1.90 (1.19 - 3.03), 0.007

*baPWV* = brachial ankle pulse wave velocity.

Model 1: adjusted for age and sex.

Model 2: model 1 + adjusted for alcohol, smoking status, physical activity, systolic blood pressure.

Model 3: model 2 + adjusted for glucose, Triglyceride, HDL cholesterole and LDL cholesterol.

Model 4: model 3 + adjusted for waist circumference.

## Discussion

We demonstrated here for the first time that both fatty liver and baPWV are associated with a CAC score > 0, independently of each other and of MetS components in a healthy Korean occupational cohort. More importantly, these results also showed that the combination of fatty liver and baPWV is associated with an increased prevalence of CAC score > 0 (1.4% in the lowest baPWV quartile with no fatty liver versus 9.8% in the highest PWV quartile with fatty liver). These results were obtained from the retrospective analysis of the data from a healthy (no prior medical history), young, occupational cohort, and the association between both fatty liver and baPWV as a continuous variable (or baPWV as a categorical variable) and CAC score > 0 persisted after adjustment for the features of MetS and life style.

To date, there are no similar studies investigating the relationships between a marker of early development of coronary artery atherosclerosis and fatty liver, baPWV and MetS features in a large, predominantly healthy, middle-aged cohort, even though some previous studies have shown associations between increased baPWV and the presence of coronary artery disease [22] or between liver fat, measured by ultrasound or CT, and CAC [23-25]. Strong associations between NAFLD and multiple complex metabolic and proinflammatory changes that have an effect on the vasculature [15] indicate that it is very difficult to identify causality from the relationship between NAFLD, PWV and CVD.

baPWV reflects the stiffness of both the central and peripheral muscular arteries and serves as a simple index of the severity of arterial stiffness and atherosclerosis [26]. Measurement of baPWV is a non-invasive and inexpensive procedure in clinical practice. To date, several studies have shown that PWV measurements obtained by non-invasive automatic devices do not only indicate vascular damage but also predict damage [27]. PWV has been associated with known cardiovascular risk factors, such as age, hypertension and diabetes mellitus [28-30]. A recently developed instrument, which measures baPWV using a volume-rendering method, has been widely used in clinical research, especially in East Asian countries during the past 10 years. PWV has also been associated with the presence and quantity of coronary artery calcium [31,32]. In this study, a receiver operating characteristic (ROC) curve was employed to define a cutoff value of baPWV that could discriminate between patients with and without a CAC score > 0, but the area under the curve was only 0.63, indicating that was nearly linear. For this reason, we believe that using a cut point to find CAC score > 0 would not be helpful in clinical practice. In the results, the prevalence of higher physical activity is significantly higher in the CAC > 0 group (*p* = 0.011). This is an unexpected finding and therefore is difficult to explain. In this cohort, information about lifetime physical activity energy expenditure is not available. We only had basic self-reported information on physical activity levels in this cohort and consequently it is likely that estimates are highly likely to be subject to measurement error and there is scope for residual confounding.

There are a few limitations to our study. Fatty liver was assessed by liver ultrasound, and ultrasonography has limited sensitivity, being unable to detect liver fat infiltration below 30% by liver weight. Ultrasonography was performed by experienced clinical radiologists who also diagnosed fatty liver based on the known standard clinical criteria that included hepatorenal echo contrast, liver brightness, and vascular blurring. We are therefore unable to include evidence of agreement between radiologists. However, in the presented analyses, we used the clinical definition of fatty liver as a dichotomous exposure variable. It is unlikely that fatty liver status was influenced by CAC score, and consequently, any random misclassification bias of fatty liver status would bias our findings (showing the association between fatty liver and CAC score > 0) toward null. Second, we used baPWV as a measurement of arterial stiffness, although it is not the gold standard method used to evaluate arterial stiffness. However, several studies have reported that baPWV has similar characteristics to carotid- femoral PWV, and thus, is widely used especially in East Asian countries [33,34]. Additionally, our study is limited to one ethnic group, and the distribution of risk factors and the association between NAFLD and CAC may differ by ethnic group.

In conclusion, we showed that both fatty liver and baPWV are independently associated with the presence of CAC, a marker of preclinical atherosclerosis. These associations are independent of conventional cardiovascular risk factors, and after exclusion of prior medical history.

## Abbreviations

baPWV: Brachial-ankle pulse wave velocity; CAD: Coronary artery disease; CAC: Coronary artery calcium; NAFLD: Nonalcoholic fatty liver disease; CVD: Cardiovascular disease; CT: Computed tomography; CV: Cardiovascular; MetS: Metabolic syndrome; HDL-C: High density lipoprotein cholesterol; BMI: Body-mass index; TG: Triglyceride; LDL-C: Low density lipoprotein cholesterol; OR: Odds ratio; CI: Confidence intervals.

## Competing interests

All authors have no competing interests.

## Authors’ contribution

KS devised hypothesis, analyzed data, wrote methods and contributed to discussion, YL devised hypothesis and wrote results and discussion, SP and SK devised hypothesis and contributed to discussion, JP, BK and JS reviewed/ edited the manuscript and contributed to discussion. All authors read and approved the final manuscript.

## Relationships with industry

All authors have no relationships with industry.
